# Acute and Delayed Effects of Post-Exercise Recovery Strategies on Explosive Performance and Markers of Muscle Damage: A Systematic Review and Network Meta-Analysis

**DOI:** 10.3390/healthcare14101321

**Published:** 2026-05-12

**Authors:** Chunlin Hou, Wenhui Yin, Fengjie Qiao

**Affiliations:** Division of Sports Science and Physical Education, Tsinghua University, Beijing 100084, China; houcl24@mails.tsinghua.edu.cn (C.H.); yinwh24@mails.tsinghua.edu.cn (W.Y.)

**Keywords:** exercise-induced muscle damage, recovery strategies, cold-water immersion, network meta-analysis

## Abstract

**Highlights:**

**What are the main findings?**

- Recovery effects after exercise-induced muscle damage were time-dependent, with most benefits observed within the first 24 h rather than at 48–72 h.
- Active recovery was most favorable for short-term countermovement jump recovery and massage for early soreness relief, and cold-water immersion showed the most consistent benefits across delayed-onset muscle soreness and creatine kinase outcomes.

**What are the implications of the main findings?**

- Post-exercise recovery strategies should be selected according to the primary goal of recovery, such as restoring explosive performance, reducing soreness, or limiting muscle-damage markers.
- For sport and clinical practice, cold-water immersion may be the most versatile option when broader short-term recovery support is needed, whereas active recovery or massage may be preferable for more specific recovery priorities.

**Abstract:**

**Background:** Exercise-induced muscle damage (EIMD) after strenuous exercise can impair neuromuscular function and increase muscle soreness. Although cold-water immersion (CWI), massage, and active recovery are widely used, their comparative effects across recovery time windows remain unclear. **Objective:** We aimed to compare and rank the effects of post-exercise physical recovery strategies on countermovement jump (CMJ), delayed-onset muscle soreness (DOMS), and creatine kinase (CK) during acute and delayed recovery. **Methods:** A systematic review and frequentist network meta-analysis were conducted in accordance with PRISMA 2020 and the PRISMA extension for network meta-analyses. Five databases (PubMed, Web of Science, SPORTDiscus, Cochrane Library, and Scopus) were searched. The study protocol and prespecified methods were publicly archived on the Open Science Framework (OSF, DOI:10.17605/OSF.IO/ASWU6). Outcomes were grouped into an acute phase (0–24 h) and a delayed phase (48–72 h). The primary analysis was restricted to the connected main network anchored by passive control. Random-effects models were used to calculate effect sizes with 95% confidence intervals, and P-scores were used to rank interventions. **Results:** In the acute phase, active recovery and CWI demonstrated the largest effect sizes for CMJ recovery at 24 h, though confidence intervals were wide. For DOMS, both CWI and massage showed beneficial effects immediately after exercise and at 24 h. CWI also reduced CK immediately after exercise and at 24 h. In the delayed phase, most effects diminished, with no significant benefit for CMJ or CK; however, CWI remained associated with lower DOMS at 48 h. **Conclusions:** Recovery effects appear to be strongly time-dependent. Active recovery may be preferable for short-term restoration of explosive performance, massage for early soreness relief, and CWI for broader short-term recovery support.

## 1. Introduction

High-intensity training and competitive sport can induce exercise-induced muscle damage (EIMD), characterised by mechanical disruption of sarcomeres, impaired excitation-contraction coupling, and a subsequent inflammatory response [1,2]. Clinically, EIMD is commonly reflected by delayed-onset muscle soreness (DOMS), elevated circulating creatine kinase (CK), and reduced neuromuscular performance [1]. Countermovement jump (CMJ) performance is frequently used to quantify this loss of explosive function [3]. Because these impairments may persist for several days, recovery strategies are widely used to reduce symptom burden and facilitate return to training and competition.

Several pairwise meta-analyses have evaluated post-exercise recovery modalities. Massage and cryotherapy have been associated with reductions in DOMS and fatigue [4], and massage has also shown favourable effects on short-term recovery of performance [5]. Cold-water immersion (CWI) has received particular attention, with previous reviews suggesting that water temperature and immersion duration may influence its efficacy [6], while other work has considered its use in team-sport settings [7]. However, conventional pairwise meta-analysis cannot simultaneously compare multiple interventions or provide a treatment hierarchy across all available options [8]. Furthermore, while previous NMAs have evaluated recovery modalities, they often aggregated outcome data across broad and heterogeneous time windows (e.g., blending immediate post-exercise effects with 48-h outcomes). This generalized pooling obscures the specific physiological trajectories of EIMD.

Recovery efficacy is also likely to be time-dependent. Acute post-exercise responses are dominated by metabolic stress and fatigue, whereas inflammatory processes and perceived soreness generally peak later, during the 24- to 48-h period [9,10]. As a result, interventions that are beneficial immediately after exercise may not retain their effects during delayed recovery. The efficacy of post-exercise recovery modalities is fundamentally tied to their underlying physiological mechanisms, which interact differently with the acute and delayed phases of recovery. In the acute phase (0–24 h), metabolic stress and the initial inflammatory cascade are predominant. Modalities such as active recovery and cold-water immersion (CWI) may directly influence these early processes. Active recovery is thought to enhance local blood flow, accelerate the clearance of metabolic by-products, and restore intracellular homeostasis, which is particularly beneficial for the rapid restoration of neuromuscular function (e.g., CMJ). Conversely, CWI induces vasoconstriction and reduces tissue temperature, thereby potentially blunting initial edema formation and the secondary injury phase. Massage, on the other hand, is widely hypothesized to modulate pain perception and local tissue stiffness, offering immediate relief from perceptual soreness. In the delayed phase (48–72 h), structural tissue repair and inflammatory resolution govern recovery, meaning that the sustained efficacy of interventions depends on whether they can persistently modulate these deeper secondary cascades.

Network meta-analysis (NMA) can integrate direct and indirect evidence within a single framework and allows the simultaneous comparison and ranking of multiple recovery interventions [8]. Therefore, the aim of this review was to evaluate the time-dependent effects of post-exercise recovery strategies on CMJ, DOMS, and CK using a frequentist NMA. To improve clinical interpretability and address network sparsity, the analyses were stratified into two recovery windows: an acute phase (0–24 h) and a delayed phase (48–72 h).

Therefore, this study aimed to compare and rank the time-dependent effects of physical recovery strategies on CMJ, DOMS, and CK using a frequentist network meta-analysis. Based on the divergent physiological mechanisms of these modalities, we hypothesized that: (1) recovery effects are highly time-dependent, with most interventions exhibiting superior efficacy in the acute phase; (2) active recovery is more advantageous for the short-term restoration of explosive performance; and (3) cold-water immersion offers broader, more sustained benefits, particularly for alleviating perceived muscle soreness (DOMS).

## 2. Materials and Methods

### 2.1. Protocol and Reporting

This systematic review and network meta-analysis was conducted and reported in accordance with the PRISMA 2020 statement and the PRISMA extension for network meta-analyses [11,12]. The review protocol and prespecified methods were publicly archived on the Open Science Framework (OSF), with the registration DOI:10.17605/OSF.IO/ASWU6. Because data extraction had already commenced at the time the protocol was finalized, the OSF record should be interpreted as a publicly archived protocol and methods record rather than a prospective registration.

### 2.2. Search Strategy and Eligibility Criteria

#### 2.2.1. Information Sources and Search Strategy

A systematic literature search was conducted on 24 January 2026, in PubMed, Web of Science, SPORTDiscus (via EBSCOhost), the Cochrane Library, and Scopus. The search strategy was developed around terms related to team-sport athletes, post-exercise physical recovery interventions, recovery-related outcomes, and randomized trial designs. Both controlled vocabulary and free-text terms were used and adapted to the syntax requirements of each database. In addition, the reference lists of relevant reviews and eligible studies were screened manually to identify potentially eligible articles not retrieved in the electronic searches. The full search strategies for all databases are provided in the Supplementary Materials.

#### 2.2.2. Eligibility Criteria

Eligibility criteria were defined according to the PICOS framework. Studies were eligible if they: (1) included team-sport athletes or healthy physically active individuals undertaking team-sport–relevant exercise protocols; (2) evaluated a clearly defined post-exercise physical recovery intervention; (3) included a passive recovery, seated rest, no-intervention control, sham intervention, or another recovery strategy as the comparator; (4) reported extractable outcome data for recovery-related outcomes, including countermovement jump (CMJ), delayed-onset muscle soreness (DOMS), creatine kinase (CK), or other relevant recovery indicators; and (5) used a randomized controlled trial or randomized crossover design. Animal studies, observational studies without a control group, studies in which nutritional supplementation was the primary intervention, conference abstracts without sufficient data, and other non-original studies were excluded.

#### 2.2.3. Study Selection

All retrieved records were imported into reference management software and duplicate records were removed before screening. Two reviewers independently screened titles and abstracts according to the predefined eligibility criteria. Full-text articles judged to be potentially eligible were then retrieved and assessed in detail. Reasons for exclusion at the full-text stage were recorded. Any disagreements between reviewers were resolved through discussion and, when necessary, consultation with a third reviewer.

### 2.3. Data Extraction and Risk of Bias

#### 2.3.1. Data Extraction

Two reviewers independently extracted data using a standardized form. Extracted information included study characteristics, participant characteristics, exercise-induced fatigue protocols, intervention and comparator details, timing of outcome assessment, and numerical outcome data at 0, 24, 48, and 72 h where available. When results were reported graphically only, numerical values were extracted using WebPlotDigitizer (version 4.6) [13]. If the required data could not be obtained directly from the published report, the study was retained for qualitative description but excluded from the relevant quantitative synthesis.

#### 2.3.2. Risk-of-Bias Assessment

Risk of bias was assessed independently by two reviewers using the Cochrane Risk of Bias 2 tool for randomized trials [14]. Disagreements were resolved through discussion and, when necessary, consultation with a third reviewer.

#### 2.3.3. Time-Window Aggregation and Subnetwork Management

To maintain physiological accuracy and avoid blurring distinct recovery trajectories, statistical analyses were inherently conducted at discrete, independent time points (i.e., exactly at 0 h, 24 h, 48 h, and 72 h post-exercise). The terms “acute recovery phase” (0–24 h) and “delayed recovery phase” (48–72 h) were utilized solely as conceptual narrative frameworks to structure the reporting of the results, rather than to mathematically pool data across disparate time points. The primary NMA was restricted to the connected main network anchored by passive control in order to preserve the transitivity assumption and avoid unstable estimates from disconnected subnetworks.

#### 2.3.4. Statistical Analysis

All analyses were conducted in R (version 4.3.2) using the netmeta (version 2.8-2) package within a frequentist framework [15,16]. Pairwise meta-analysis and network meta-analysis were performed, where appropriate, to compare competing recovery interventions. Standardized mean differences (SMDs) with 95% confidence intervals were used for outcomes measured on different scales, whereas mean differences (MDs) were used when the same scale was applied across studies [17]. Random-effects models were prespecified because of anticipated clinical heterogeneity [18]. P-scores were used to rank interventions [19]. Heterogeneity was assessed using I^2^ [20], and local inconsistency and small-study effects were explored using node-splitting and comparison-adjusted funnel plots [16]. Where quantitative synthesis was not feasible, findings were summarized narratively.

## 3. Results

### 3.1. Study Selection and Characteristics

**Figure 1:**
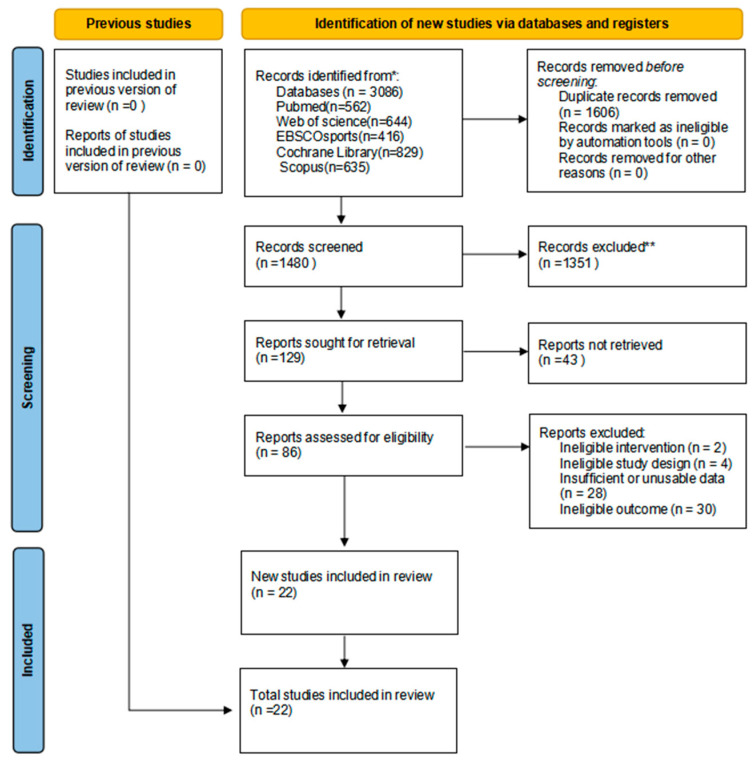
PRISMA flow diagram of study selection. * Databases searched include PubMed, Web of Science, SPORTDiscus, Cochrane Library, and Scopus. ** Records excluded by human reviewers based on title and abstract screening.

The study selection process is shown in Figure 1. A total of 3086 records were identified through database searching. After duplicate removal, 1480 records remained for title and abstract screening. Eighty-six full-text articles were assessed for eligibility, of which 64 were excluded for predefined reasons (see Supplementary Table S2).

Ultimately, 22 randomized studies were included in the review and network meta-analysis. The characteristics of the included studies are summarized in Table 1, and the main network meta-analysis findings are summarized in Table 2.

**Figure 2:**
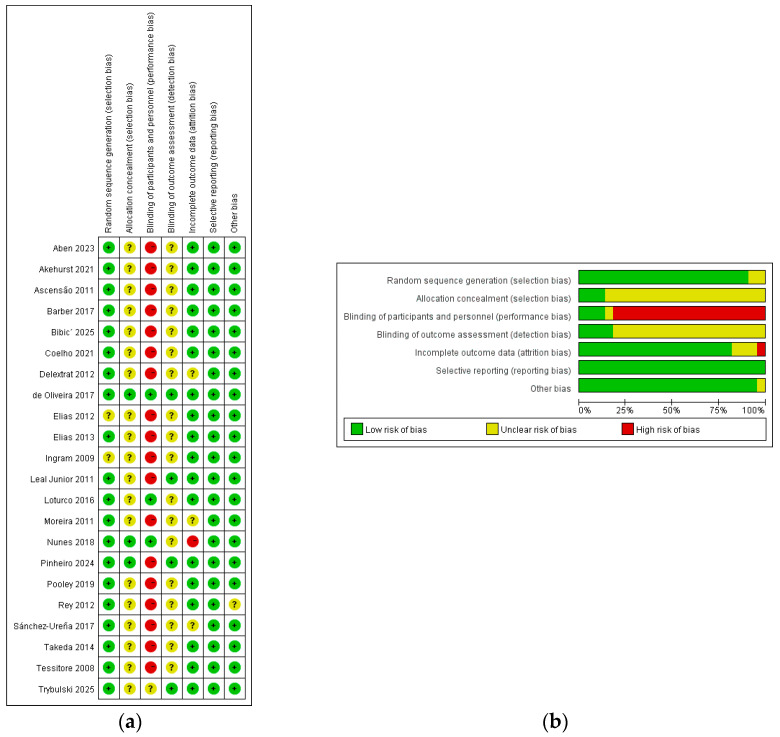
Risk-of-bias summary. (**a**) Risk-of-bias summary plot. (**b**) Risk-of-bias traffic light plot. Green (+) indicates a low risk of bias, yellow (?) indicates an unclear risk of bias, and red (−) indicates a high risk of bias. The corresponding reference citations for the studies presented in the figure are as follows: Aben 2023 [21], Akehurst 2021 [33], Ascensão 2011 [25], Barber 2017 [42], Bibić 2025 [29], Coelho 2021 [28], Delextrat 2012 [24], de Oliveira 2017 [22], Elias 2012 [31], Elias 2013 [32], Ingram 2009 [34], Leal Junior 2011 [30], Loturco 2016 [35], Moreira 2011 [23], Nunes 2018 [38], Pinheiro 2024 [41], Pooley 2019 [40], Rey 2012 [37], Sánchez-Ureña 2017 [27], Takeda 2014 [36], Tessitore 2008 [26], and Trybulski 2025 [39].

Risk-of-bias judgments are summarized in Figure 2. The included studies covered a range of exercise protocols, including soccer-specific exercise, rugby-related workloads, resistance exercise, and prolonged endurance running, and participants ranged from recreationally active individuals to elite athletes.

### 3.2. Network Geometry

**Figure 3:**
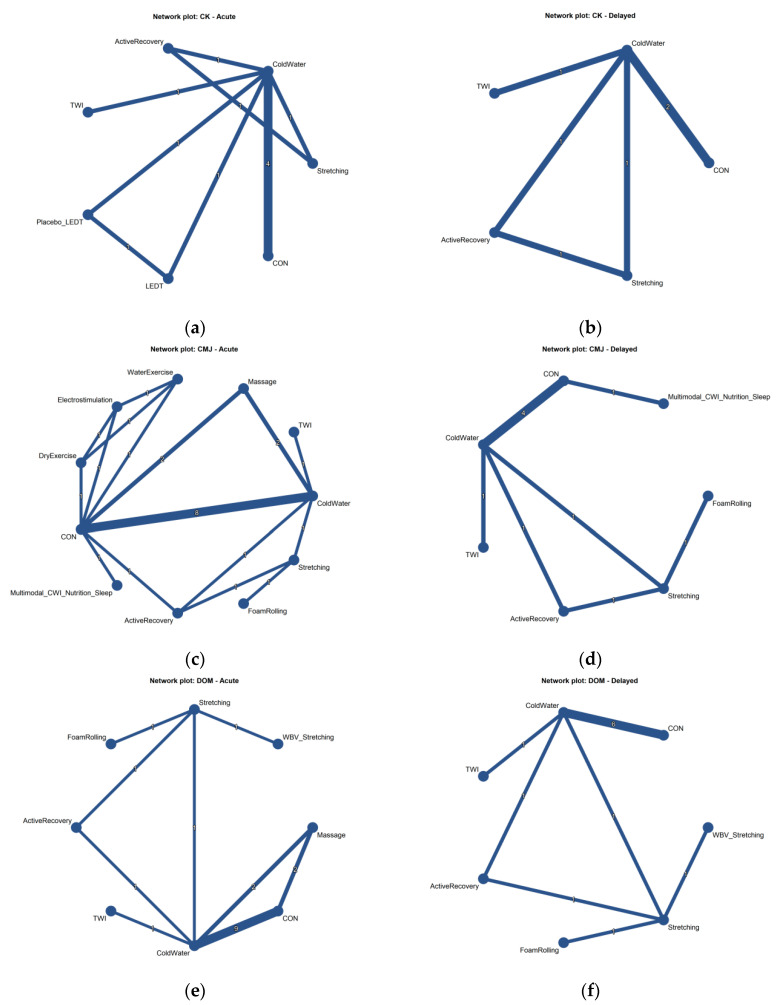
Network plots for each outcome and recovery window. (**a**) Creatine kinase (CK) in the acute phase; (**b**) CK in the delayed phase; (**c**) Countermovement jump (CMJ) in the acute phase; (**d**) CMJ in the delayed phase; (**e**) Delayed-onset muscle soreness (DOMS) in the acute phase; and (**f**) DOMS in the delayed phase. Node size is proportional to the total number of participants, and edge thickness is proportional to the number of direct comparisons. The numbers on the lines connecting the nodes represent the exact number of direct comparison studies between the respective interventions. CON = passive control; CWI = cold-water immersion.

Connected intervention networks were constructed for both the acute and delayed recovery periods, each anchored by passive control. The largest amount of direct evidence linked passive control with CWI and massage (Figure 3).

### 3.3. Acute Recovery Phase (0–24 h)

No significant differences in CMJ were observed immediately after exercise. At 24 h, active recovery showed the largest benefit over control (SMD = 0.944, 95% CI 0.201 to 1.686), followed by CWI (SMD = 0.447, 95% CI 0.124 to 0.770).

For DOMS, both CWI and massage showed significant analgesic effects immediately after exercise and at 24 h. At 0 h, CWI produced an SMD of −1.147 (95% CI −2.106 to −0.188), while massage produced an SMD of −2.237 (95% CI −3.995 to −0.479). At 24 h, the effects were larger for both CWI (SMD = −2.187, 95% CI −3.193 to −1.182) and massage (SMD = −2.616, 95% CI −4.427 to −0.804).

For CK, CWI was the only intervention associated with a significant reduction in the acute phase, both immediately after exercise (SMD = −0.494, 95% CI −0.944 to −0.044) and at 24 h (MD = −177.100, 95% CI −343.253 to −10.946).

**Figure 4:**
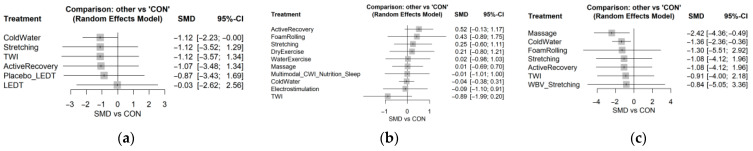
Forest plots for the acute recovery phase (0–24 h). Relative effects of post-exercise recovery strategies versus passive control (CON) during the acute recovery phase. Panel (**a**): creatine kinase (CK); Panel (**b**): countermovement jump (CMJ); Panel (**c**): delayed-onset muscle soreness (DOMS). Data are shown as standardized mean differences (SMDs) or mean differences (MDs) with 95% confidence intervals. Positive values favour better CMJ recovery, whereas negative values favour lower CK or DOMS. CWI = cold-water immersion; TWI = thermoneutral water immersion; CON = passive control.

According to the P-score rankings, active recovery ranked highest for CMJ restoration, massage ranked highest for DOMS reduction, and CWI ranked highest for CK reduction during the acute phase (Figure 4).

### 3.4. Delayed Recovery Phase (48–72 h)

**Figure 5:**
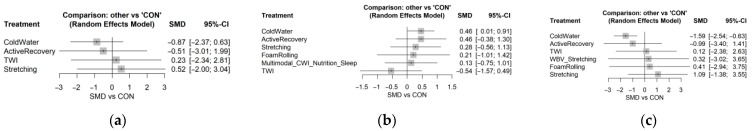
Forest plots for the delayed recovery phase (48–72 h). Relative effects of post-exercise recovery strategies versus passive control (CON) during the delayed recovery phase. Panel (**a**): creatine kinase (CK); Panel (**b**): countermovement jump (CMJ); Panel (**c**): delayed-onset muscle soreness (DOMS). Data are shown as standardized mean differences (SMDs) or mean differences (MDs) with 95% confidence intervals. Positive values favour better CMJ recovery, whereas negative values favour lower CK or DOMS. CWI = cold-water immersion; TWI = thermoneutral water immersion; CON = passive control.

By 48 h, treatment effects had attenuated substantially. No intervention retained a significant advantage over control for CMJ or CK. However, CWI remained the only intervention associated with lower DOMS at 48 h (SMD = −1.568, 95% CI −2.578 to −0.558) (Figure 5).

### 3.5. Heterogeneity and Inconsistency

Node-splitting analyses did not identify statistically significant disagreement between direct and indirect evidence in evaluable loops. Comparison-adjusted funnel plots appeared broadly symmetrical. Nevertheless, substantial global heterogeneity was present in the DOMS and CK networks (I^2^ > 75%).

## 4. Discussion

### 4.1. Principal Findings

This review indicates that the comparative efficacy of post-exercise physical recovery strategies is highly time-dependent and outcome-specific. Across the networks, the clearest benefits were concentrated within the first 24 h after exercise, whereas most effects were attenuated by 48–72 h. This pattern is clinically important because it suggests that recovery interventions are most relevant when athletes face short turnarounds between training sessions or competition, rather than when sufficient time is available for spontaneous recovery.

Although specific Minimal Clinically Important Difference (MCID) thresholds were not formally established across all diverse athlete populations, the magnitude of the observed effects provides clinical context. For example, active recovery yielded a large effect size (SMD > 0.8) for CMJ at 24 h, whereas the analgesic effects of massage and CWI on DOMS also reached large magnitudes (SMD < −1.0). Despite these large point estimates, the wide confidence intervals necessitate a cautious interpretation. The findings also show that recovery modalities should not be treated as interchangeable. Active recovery showed the most favourable short-term restoration of explosive performance, massage was associated with the largest short-term reductions in perceived soreness, and cold-water immersion (CWI) produced the most consistent overall profile by benefiting soreness and biochemical markers across the acute period and remaining beneficial for DOMS at 48 h. Taken together, these results support a more selective model of recovery prescription in which the intervention is matched to the recovery target, the time available before the next performance demand, and the biological phase of recovery.

### 4.2. Mechanistic Interpretation

The apparent advantage of active recovery for CMJ at 24 h may reflect mechanisms that are more closely related to restoration of neuromuscular function than to suppression of muscle-damage processes per se. Low-intensity movement performed after strenuous exercise may enhance muscle perfusion, accelerate metabolite clearance, and help restore intracellular homeostasis, thereby improving readiness for tasks that depend on rapid force production [43,44]. In practical terms, this may explain why active recovery appears to support explosive performance despite not showing the same breadth of effects on soreness or CK as CWI.

By contrast, massage and CWI appear to act more strongly on structural-inflammatory and perceptual pathways. Massage has been proposed to influence inflammatory signalling, local circulation, and pain modulation [45,46], which is consistent with its favourable effects on DOMS in the present review. CWI may reduce tissue temperature, metabolic demand, vascular permeability, and oedema formation [47,48], thereby acting across several pathways relevant to both soreness and secondary muscle damage. The divergence between performance recovery and symptom/biochemical recovery in the present results reinforces the idea that no single marker should be taken as a complete surrogate for recovery status. An athlete may report less soreness yet not fully recover explosive function, or recover performance while biochemical disturbance remains detectable.

### 4.3. Delayed Recovery and the Role of CWI

The marked attenuation of effects beyond 48 h suggests that the later stages of recovery are increasingly governed by endogenous repair processes rather than by strong continued treatment effects. Once the immediate metabolic and perceptual consequences of exercise subside, the trajectory of recovery is likely to depend more on tissue repair, inflammatory resolution, sleep, nutrition, and subsequent load management than on a single post-exercise modality [49]. This may explain why most interventions no longer retained a significant advantage over control for CMJ or CK in the delayed phase.

The trajectory of delayed recovery is multifactorial. Once the immediate metabolic consequences of exercise subside, recovery depends not only on physical modalities but also heavily on psychophysiological variables, such as stress management and sleep quality. For instance, recent research highlights that stress-related sleep disturbances can significantly impair performance and prolong recovery cascades in athletic populations [50]. Therefore, physical interventions like CWI should ideally be integrated with broader behavioral and sleep hygiene strategies.

CWI was the only intervention that maintained a significant effect on DOMS at 48 h, which may indicate more than a transient analgesic effect [9,51]. A plausible interpretation is that CWI attenuated part of the secondary cascade that contributes to prolonged soreness, possibly by modulating oedema formation or inflammatory amplification in the early post-exercise period. At the same time, the persistence of a DOMS benefit without parallel sustained effects on CMJ or CK highlights an important conceptual point: delayed soreness, neuromuscular recovery, and biochemical leakage do not necessarily recover at the same rate or respond similarly to the same intervention. Accordingly, practitioners should be cautious about inferring global recovery from a single positive change in one outcome domain.

### 4.4. Clinical Implications

These findings support a targeted and time-sensitive approach to recovery prescription in team-sport settings. For sports physiotherapists, team medical staff, and strength and conditioning practitioners, the choice of recovery modality should be guided by the primary short-term objective rather than by routine habit. If the immediate priority is restoration of explosive performance before the next session or match, active recovery may be the most appropriate option. If the priority is rapid symptom relief, massage appears useful for short-term reduction in soreness. When a broader recovery objective is required across soreness and muscle-damage markers, particularly over the first 24–48 h, CWI may be the most versatile strategy.

The results are also relevant for fixture congestion and tournament play. In schedules with less than 24 h between demanding efforts, even modest gains in perceptual recovery or neuromuscular readiness may have operational value. In contrast, when 48 h or longer is available before the next important performance demand, the additional benefit of many interventions may be limited, and simpler or less resource-intensive strategies may be sufficient. This distinction matters for implementation because staff time, equipment availability, athlete preference, and travel constraints often determine what is feasible in real-world environments.

Another practical implication is that intervention selection should be individualized. Athletes differ in their soreness response, competition role, training age, and tolerance for cold exposure or massage. The present findings are therefore most useful as a decision framework rather than as a universal protocol. Practitioners may combine the evidence from this review with monitoring data, athlete feedback, and contextual demands to select the most appropriate recovery strategy on a case-by-case basis.

At the same time, recovery interventions should not be selected solely on the basis of short-term symptom reduction. Post-exercise inflammation is also involved in tissue remodelling and long-term adaptation [52]. Repeated use of CWI after training may attenuate anabolic signalling and reduce longer-term gains in muscle strength and mass [53]. From a periodization perspective, this means that the most effective acute recovery intervention may not always be the most appropriate chronic strategy. During congested competition periods, rapid restoration of readiness may justifiably take precedence. During training blocks aimed at maximizing adaptation, however, frequent use of modalities that blunt inflammatory or anabolic responses should be considered more cautiously. The optimal recovery strategy is therefore context-dependent and should reflect whether the primary goal is next-day performance, symptom control, or long-term physiological adaptation.

Furthermore, it is increasingly evident that recovery protocols cannot be applied uniformly across all populations. Recent evidence highlights that recovery kinetics can vary significantly based on individual factors such as gender, age, and sport type. For instance, recent studies indicate that structural and functional recovery trajectories following strenuous exercise exhibit distinct, sex-specific patterns, highlighting that men and women may experience different intermuscular compensations during the delayed recovery phase [54]. Similarly, chronological age heavily influences the systemic inflammatory response and subsequent tissue repair, with older skeletal muscle often displaying delayed and prolonged recovery kinetics [55]. Consequently, the hierarchical rankings provided in this network meta-analysis should not be viewed as a “one-size-fits-all” prescription. Future applications of these findings must be contextualized within the athlete’s specific demographic profile and competitive environment.

### 4.5. Limitations

Several limitations must be acknowledged. First, although we grouped follow-up time points into broader conceptual windows (acute and delayed phases) to facilitate clinical interpretation, the network geometry remained sparse for certain modalities (e.g., foam rolling or thermoneutral water immersion). This sparsity limited statistical power, meaning that the absence of statistical inconsistency does not guarantee true network consistency. Second, due to this network sparsity and the substantial heterogeneity (I^2^ > 75%) inherent in the DOMS and CK networks, a formal certainty-of-evidence assessment (such as CINeMA or GRADE for NMA) could not be reliably computed. The calculated rankings (P-scores) should therefore be interpreted with caution and not viewed as definitive proof of superiority. Third, the included studies exhibited considerable clinical heterogeneity, encompassing different exercise protocols, participant levels (from youth to elite), and intervention doses (e.g., varying water temperatures and immersion durations). This heterogeneity, combined with the small sample sizes of many primary studies, challenges the transitivity assumption and limits broad generalizability. Fourth, due to these limited study pools, we could not perform robust sensitivity analyses excluding studies with high risk of bias, nor could we conduct formal statistical tests for publication bias (e.g., Egger’s test) beyond visual inspection of funnel plots. Fifth, we did not anchor our findings to Minimal Clinically Important Differences (MCID), which means statistically significant effect sizes (SMDs) may not directly translate to practically meaningful improvements for every athlete. Finally, it should be noted that the study protocol was archived retrospectively (after data extraction had commenced), which deviates from optimal prospective registration standards. Furthermore, changes in creatine kinase (CK) were aggregated using mean differences (MDs) under the assumption of comparable absolute assay units (U/L) across the specific included trials in that subnetwork. However, utilizing standardized mean differences (SMDs) may have more robustly accounted for the inherent variability in biochemical assay ranges across different laboratories.

## 5. Conclusions

The comparative efficacy of post-exercise physical recovery strategies appears to be strongly time-dependent. Within 24 h after exercise, active recovery ranked highest for the restoration of explosive performance, massage was associated with the largest short-term relief of perceived soreness, and CWI presented a broadly favorable profile. Beyond 48 h, the additional benefits of most recovery strategies are markedly reduced, suggesting that natural recovery processes may be sufficient when there is no immediate performance demand.

## Figures and Tables

**Table 1:** Characteristics of included studies.

Follow-Up	Outcomes	Recovery Arms	Exercise Protocol	Participants	Study
Baseline, 24 h, 48 h	CMJ	Multimodal recovery (10 min CWI + nutrition + sleep hygiene); passive control	High-intensity field session with repeated running, collisions, and high-intensity efforts	10 male collegiate rugby players	Aben et al. (2023) [21]
Baseline, 0 h, 24 h, 48 h, 72 h	DOMS	PBMT 100/200/400 mW; placebo PBMT	Isokinetic eccentric knee extension (75 reps at 60°/s)	28 professional male soccer players	de Oliveira et al. (2017) [22]
Baseline, 0 h, 24 h	CMJ, DOMS	CWI (12 min, 15 ± 1 °C); passive recovery	Futsal match	10 professional male futsal players	Moreira et al. (2015) [23]
Baseline, 0 h, 24 h	CMJ, DOMS	CWI (10 min, 11 ± 0.7 °C); massage (30 min); passive recovery	Competitive basketball match	16 university basketball players (men and women)	Delextrat et al. (2013) [24]
Baseline, 0 h, 24 h, 48 h	CMJ, DOMS, CK	CWI (10 min, 10 °C); TWI (10 min, 35 °C)	Official soccer match (90 min)	20 elite male soccer players	Ascensão et al. (2011) [25]
Baseline, 0 h	CMJ	Water exercise; dry exercise; electrostimulation; seated rest	Standard futsal match	10 male futsal players	Tessitore et al. (2008) [26]
Baseline, 0 h, 24 h, 48 h	CMJ, DOMS	Continuous CWI; intermittent CWI; passive control	Three 90 min basketball technical-tactical training sessions	10 male youth basketball players	Sánchez-Ureña et al. (2017) [27]
Baseline, 0 h, 24 h, 48 h	DOMS	CWI (10 min, 10 °C); bioceramic garment; passive rest	One-off friendly soccer match	25 university-level soccer players	Coelho et al. (2021) [28]
Baseline, 0 h, 24 h, 48 h	CMJ, DOMS	Foam rolling (20 min); static stretching (20 min)	Football match	20 elite U-17 soccer players	Bibić et al. (2025) [29]
0 h, 24 h	CK	Active LEDT; placebo LEDT; CWI (5 min, 5 °C)	Three Wingate tests	6 elite male futsal players	Leal Junior et al. (2011) [30]
Baseline, 0 h, 24 h, 48 h	CMJ, DOMS	CWI (14 min, 12 °C); passive rest	Standardized Australian football training	14 male professional Australian football players	Elias et al. (2012) [31]
Baseline, 0 h, 24 h, 48 h	CMJ, DOMS	CWI (14 min, 12 °C); contrast water therapy; passive recovery	Practice match (75 min)	24 elite male Australian football players	Elias et al. (2013) [32]
0 h, 24 h, 48 h, 72 h	DOMS	Static stretching; WBV + stretching	4 × 10 eccentric knee extensions at 60% 1RM; final set to failure	22 elite field hockey players (14 men, 8 women)	Akehurst et al. (2021) [33]
Baseline, 0 h, 24 h, 48 h	DOMS, CK	CWI (15 min, 10 °C); contrast water therapy; control	80 min simulated team-sport protocol + 20 m shuttle run to exhaustion	11 men with team-sport experience	Ingram et al. (2009) [34]
Baseline, 0 h, 24 h, 48 h, 72 h	CMJ, DOMS, CK	Far-infrared-emitting clothing; placebo clothing	100 drop jumps from 45 cm (6 s intervals)	21 elite male soccer players	Loturco et al. (2016) [35]
Baseline, 0 h, 24 h	CMJ, CK	CWI (10 min, 15 °C); passive rest	80 min simulated rugby protocol	20 trained male collegiate rugby players	Takeda et al. (2014) [36]
Baseline, 24 h	CMJ	Active recovery (20 min); passive recovery	Standardized soccer training (45 min)	31 professional male soccer players	Rey et al. (2012) [37]
Baseline, 24 h, 48 h	CMJ	Bioceramic garment; placebo garment	Two-week preseason high-intensity training program	20 elite male futsal players	Nunes et al. (2020) [38]
Baseline, 0 h, 24 h, 48 h, 72 h	CK	Cold + intermittent compression; ice only; sham/placebo	Box jumps from 50 cm	45 amateur male soccer players	Trybulski et al. (2025) [39]
Baseline, 0 h, 48 h	CMJ, DOMS, CK	Static stretching; active recovery; CWI (10 min, 14 ± 0.8 °C)	Formal 80 min match	15 elite male youth soccer players	Pooley et al. (2020) [40]
Baseline, 24 h	CMJ, CK	CWI (10 min, 10 ± 1 °C); passive rest	Nine-day preseason microcycle	23 U20 semi-professional male soccer players	Pinheiro et al. (2024) [41]
Baseline, 0 h, 24 h, 48 h	CMJ, DOMS, CK	CWI (2 × 5 min, 10 °C); control	Bath University Rugby Shuttle Test (BURST)	16 club-level male rugby players	Barber et al. (2020) [42]

Note. CMJ = countermovement jump; DOMS = delayed-onset muscle soreness; CK = creatine kinase; CWI = cold-water immersion; TWI = thermoneutral water immersion; PBMT = photobiomodulation therapy; LEDT = light-emitting diode therapy; WBV = whole-body vibration; 1RM = 1-repetition maximum.

**Table 2:** Summary of main network meta-analysis findings across outcomes and recovery windows.

Outcome	Recovery Window/Time Point	Highest-Ranked Intervention (By P-Score)	Effect Versus Control	Interpretation
CMJ	Acute phase (24 h)	Active recovery	SMD = 0.944 (95% CI 0.201 to 1.686)	Largest improvement in explosive performance versus control
CMJ	Acute phase (24 h)	Cold-water immersion (CWI)	SMD = 0.447 (95% CI 0.124 to 0.770)	Moderate benefit for CMJ recovery versus control
DOMS	Acute phase (0 h)	Massage	SMD = −2.237 (95% CI −3.995 to −0.479)	Strong immediate reduction in perceived soreness
DOMS	Acute phase (0 h)	Cold-water immersion (CWI)	SMD = −1.147 (95% CI −2.106 to −0.188)	Significant immediate soreness reduction
DOMS	Acute phase (24 h)	Massage	SMD = −2.616 (95% CI −4.427 to −0.804)	Strong reduction in soreness at 24 h
DOMS	Acute phase (24 h)	Cold-water immersion (CWI)	SMD = −2.187 (95% CI −3.193 to −1.182)	Large reduction in soreness at 24 h
CK	Acute phase (0 h)	Cold-water immersion (CWI)	SMD = −0.494 (95% CI −0.944 to −0.044)	Lower CK immediately after exercise
CK	Acute phase (24 h)	Cold-water immersion (CWI)	MD = −177.100 (95% CI −343.253 to −10.946)	Lower CK at 24 h
CMJ	Delayed phase (48–72 h)	None	No significant effect versus control	Most effects attenuated beyond 24 h
CK	Delayed phase (48–72 h)	None	No significant effect versus control	Most effects attenuated beyond 24 h
DOMS	Delayed phase (48 h)	Cold-water immersion (CWI)	SMD = −1.568 (95% CI −2.578 to −0.558)	Only sustained protective effect in the delayed phase

Table note. Positive SMD values indicate better CMJ recovery. Negative SMD or MD values indicate lower DOMS or CK relative to control. CMJ = countermovement jump; DOMS = delayed-onset muscle soreness; CK = creatine kinase; CWI = cold-water immersion; MD = mean difference; SMD = standardized mean difference.

## Data Availability

The data presented in this study are available in the article.

## Supplementary Materials

The following supporting information can be downloaded at: https://www.mdpi.com/article/10.3390/healthcare14101321/s1, Figure S1: Comparison-adjusted funnel plots for each outcome and recovery window; Figure S2: League heatmaps for each outcome and recovery window; Figure S3: Netheat plot for the CMJ network in the acute recovery phase; Figure S4: Node-splitting analyses for acute recovery networks; Table S1: Full search strategies; Table S2: Full-text articles excluded after eligibility assessment; Tables S3–S8: League tables for network meta-analysis results across outcomes and recovery windows; Table S9: The PRISMA 2020 reporting checklist.

## Abbreviations

The following abbreviations are used in this manuscript:

CK	Creatine kinase
CMJ	Countermovement jump
CWI	Cold-water immersion
DOMS	Delayed-onset muscle soreness
OSF	Open Science Framework
LEDT	Light-emitting diode therapy
PBMT	Photobiomodulation therapy
TWI	Thermoneutral water immersion
WBV	Whole-body vibration
