# Supplementary material for: Acute and Delayed Effects of Post-Exercise Recovery Strategies on Explosive Performance and Markers of Muscle Damage: A Systematic Review and Network Meta-Analysis
Source: Healthcare (Basel). 2026 May 12;14(10):1321. doi: 10.3390/healthcare14101321 (PMC13206571; doi:10.3390/healthcare14101321)
Supplement: Supplementary file 1 [file healthcare-14-01321-s001.zip › healthcare-4256741-supplementary.pdf]

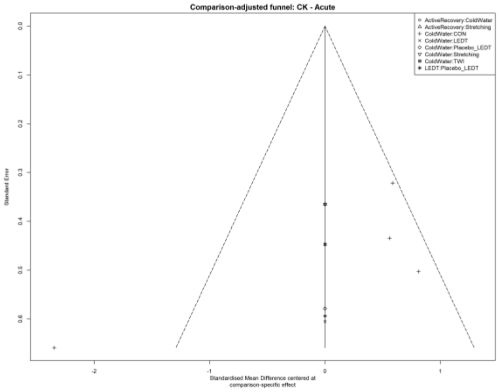
Comparison-adjusted funnel: CK - Acute
ActiveRecovery:ColdWater
ActiveRecovery:Stretching
ColdWater:CON
ColdWater:LEDT
ColdWater:Placebo_LEDT
ColdWater:Stretching
ColdWater:TWI
LEDT:Placebo_LEDT
Standard Error
Standardised Mean Difference centered at comparison-specific effect

(**a**)

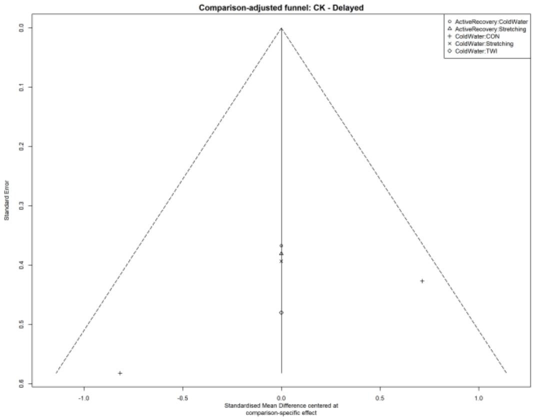
Comparison-adjusted funnel: CK - Delayed
ActiveRecovery:ColdWater
ActiveRecovery:Stretching
ColdWater:CON
ColdWater:Stretching
ColdWater:TWI
Standard Error
Standardised Mean Difference centered at comparison-specific effect

(**b**)

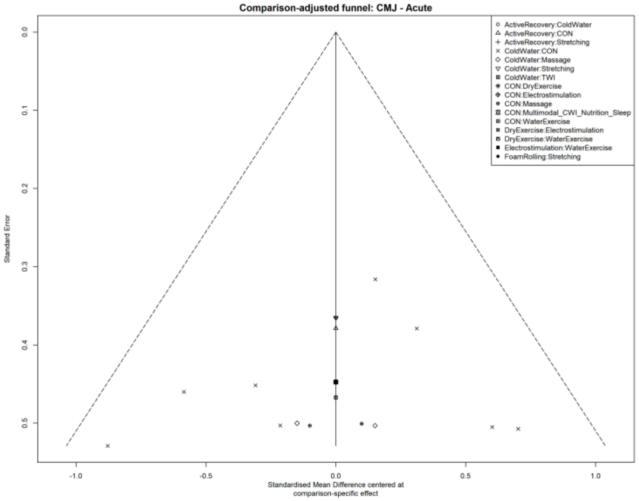
Comparison-adjusted funnel: CMJ - Acute
ActiveRecovery:ColdWater
ActiveRecovery:CON
ActiveRecovery:Stretching
ColdWater:CON
ColdWater:Massage
ColdWater:Stretching
ColdWater:TWI
CON:DryExercise
CON:Electrostimulation
CON:Massage
CON:Multimodal_CWI_Nutrition_Sleep
CON:WaterExercise
DryExercise:Electrostimulation
DryExercise:WaterExercise
Electrostimulation:WaterExercise
FoamRolling:Stretching
Standard Error
Standardised Mean Difference centered at comparison-specific effect

(c)

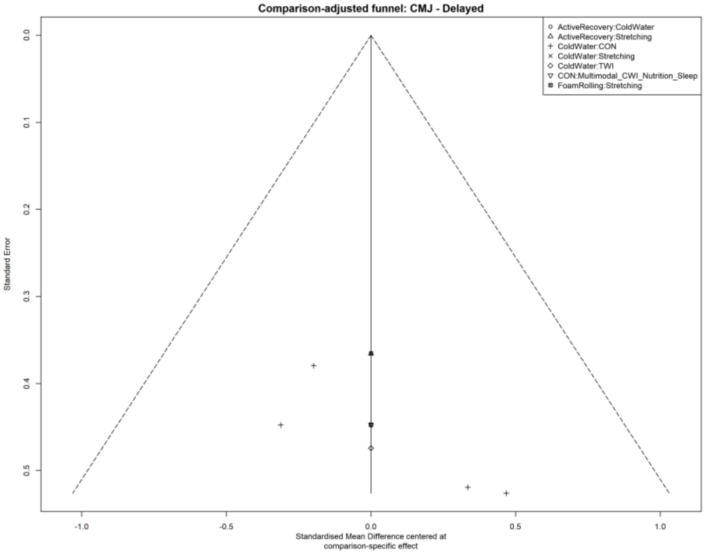
Comparison-adjusted funnel: CMJ - Delayed
ActiveRecovery:ColdWater
ActiveRecovery:Stretching
ColdWater:CON
ColdWater:Stretching
ColdWater:TWI
CON:Multimodal_CWI_Nutrition_Sleep
FoamRolling:Stretching
Standard Error
Standardised Mean Difference centered at comparison-specific effect

(d)

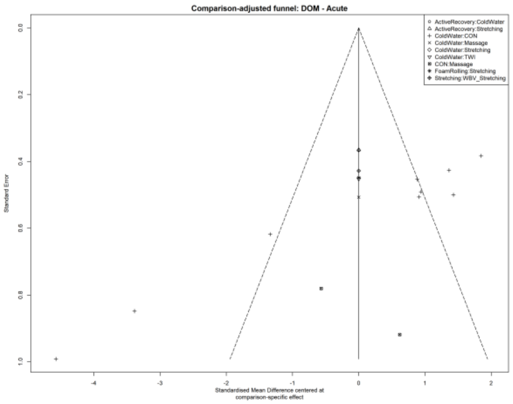

(e)

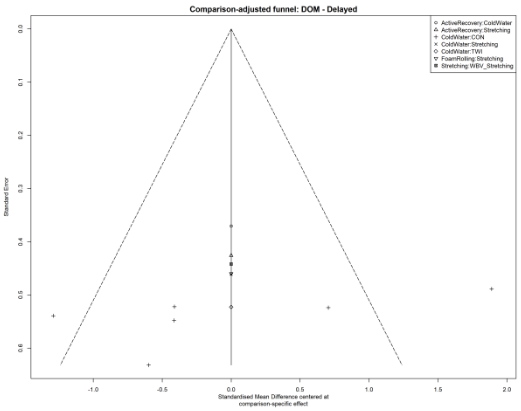

(f)

Figure S1. Comparison-adjusted funnel plots for each outcome and recovery window.
(A) CK, acute phase; (B) CK, delayed phase; (C) CMJ, acute phase; (D) CMJ, delayed phase; (E) DOMS, acute phase; and (F) DOMS, delayed phase.

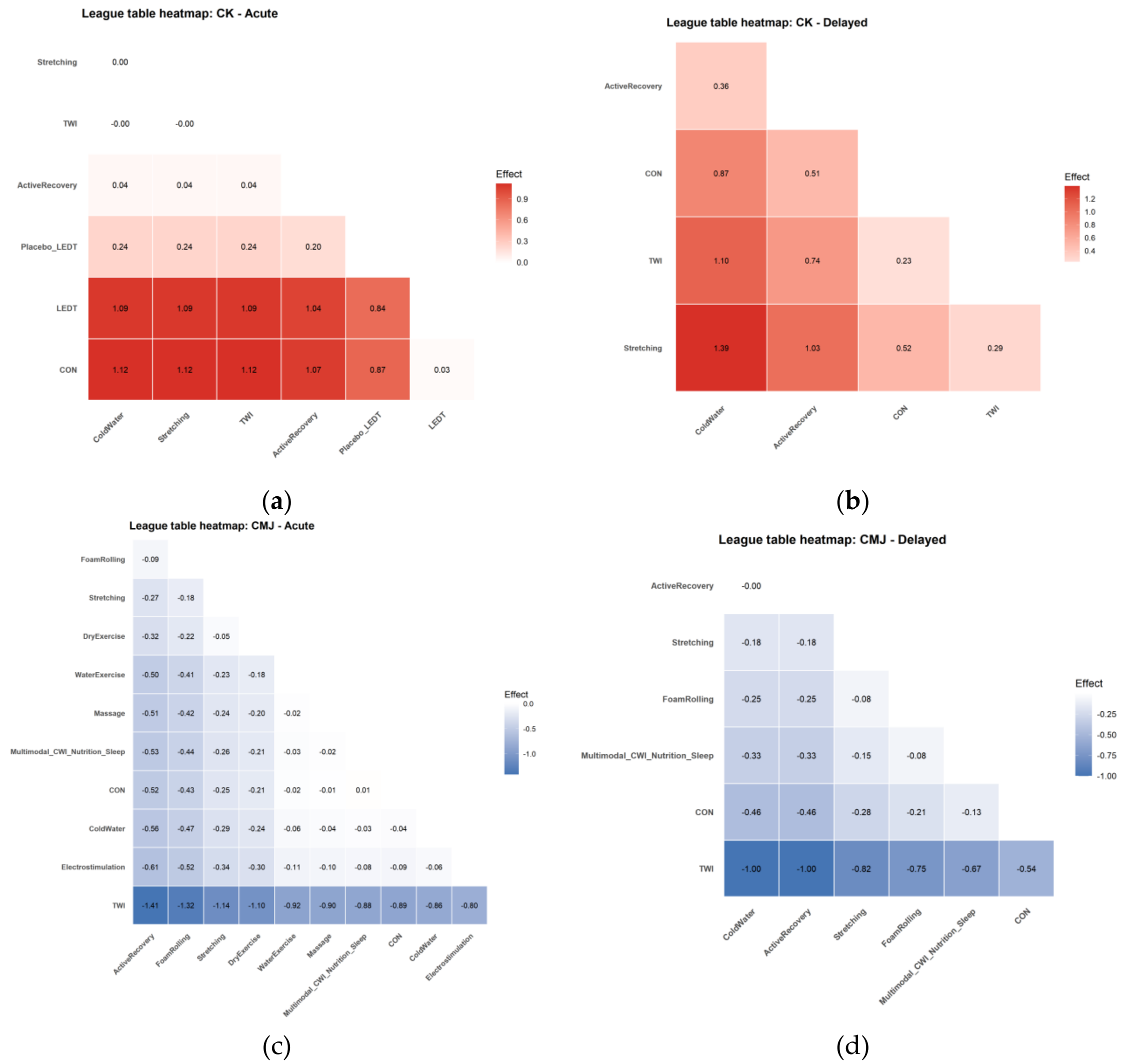
League table heatmap: CK - Acute
Stretching 0.00
TWI -0.00 -0.00
ActiveRecovery 0.04 0.04 0.04
Placebo_LEDT 0.24 0.24 0.24 0.20
LEDT 1.09 1.09 1.09 1.04 0.84
CON 1.12 1.12 1.12 1.07 0.87 0.03
ColdWater
Stretching
TWI
ActiveRecovery
Placebo_LEDT
LEDT
Effect
0.9
0.6
0.3
0.0
(a)
League table heatmap: CK - Delayed
ActiveRecovery 0.36
CON 0.87 0.51
TWI 1.10 0.74 0.23
Stretching 1.39 1.03 0.52 0.29
ColdWater
ActiveRecovery
CON
TWI
Effect
1.2
1.0
0.8
0.6
0.4
(b)
League table heatmap: CMJ - Acute
FoamRolling -0.09
Stretching -0.27 -0.18
DryExercise -0.32 -0.22 -0.05
WaterExercise -0.50 -0.41 -0.23 -0.18
Massage -0.51 -0.42 -0.24 -0.20 -0.02
Multimodal_CWI_Nutrition_Sleep -0.53 -0.44 -0.26 -0.21 -0.03 -0.02
CON -0.52 -0.43 -0.25 -0.21 -0.02 -0.01 0.01
ColdWater -0.56 -0.47 -0.29 -0.24 -0.06 -0.04 -0.03 -0.04
Electrostimulation -0.61 -0.52 -0.34 -0.30 -0.11 -0.10 -0.08 -0.09 -0.06
TWI -1.41 -1.32 -1.14 -1.10 -0.92 -0.90 -0.88 -0.89 -0.86 -0.80
ActiveRecovery
FoamRolling
Stretching
DryExercise
WaterExercise
Massage
Multimodal_CWI_Nutrition_Sleep
CON
ColdWater
Electrostimulation
Effect
0.0
-0.5
-1.0
(c)
League table heatmap: CMJ - Delayed
ActiveRecovery -0.00
Stretching -0.18 -0.18
FoamRolling -0.25 -0.25 -0.08
Multimodal_CWI_Nutrition_Sleep -0.33 -0.33 -0.15 -0.08
CON -0.46 -0.46 -0.28 -0.21 -0.13
TWI -1.00 -1.00 -0.82 -0.75 -0.67 -0.54
ColdWater
ActiveRecovery
Stretching
FoamRolling
Multimodal_CWI_Nutrition_Sleep
CON
Effect
-0.25
-0.50
-0.75
-1.00
(d)

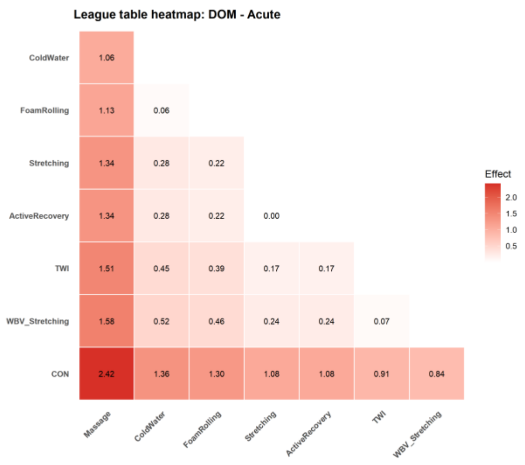

(e)

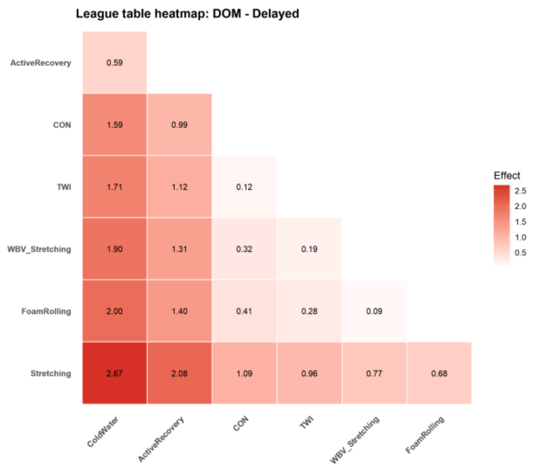

(f)

Figure S2. League heatmaps for each outcome and recovery window.
(A) CK, acute phase; (B) CK, delayed phase; (C) CMJ, acute phase; (D) CMJ, delayed phase; (E) DOMS, acute phase; and (F) DOMS, delayed phase.

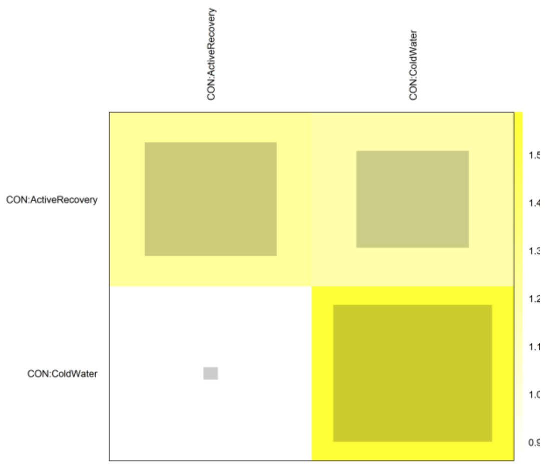

Figure S3. Netheat plot for the CMJ network in the acute recovery phase.

The netheat plot illustrates the contribution of individual direct comparisons to network inconsistency in the acute CMJ network. Warmer colors indicate greater inconsistency contribution from specific comparisons or loops within the network.

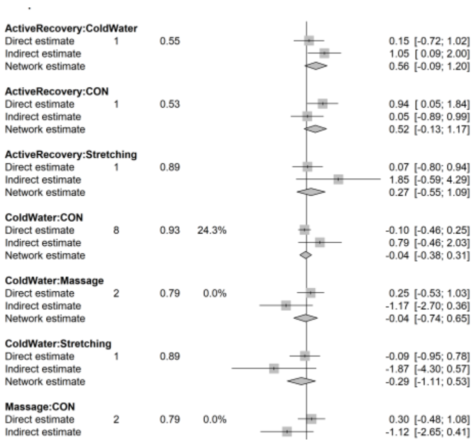

(a)

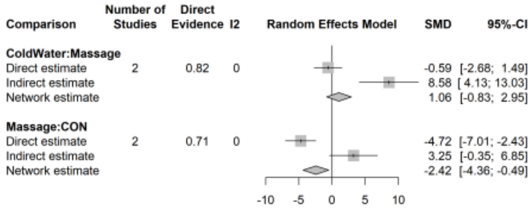

(b)

Figure S4. Node-splitting analyses for acute recovery networks.

Node-splitting analyses were performed to compare direct and indirect evidence in networks where these analyses were estimable. (A) CMJ in the acute recovery phase; (B) DOMS in the acute recovery phase.

Supplementary Table S1. Full search strategies.

Electronic database searches used for the systematic review of post-exercise physical recovery interventions in team-sport athletes.

Searches were conducted on January 24, 2026. Search syntax was adapted to the indexing rules and operators of each database. The number of records retrieved from each database before deduplication is shown in the final column.

Database	Platform	Search date	Full search strategy	Records retrieved
**PubMed**	**PubMed**	**January 24, 2026**	**("soccer"[tiab] OR "football"[tiab] OR "basketball"[tiab] OR "volleyball"[tiab] OR "rugby"[tiab] OR "handball"[tiab] OR "hockey"[tiab] OR "lacrosse"[tiab] OR "team sport*"[tiab] OR "team game*"[tiab] OR "futsal"[tiab])<br>AND<br>("recovery"[tiab] OR "cool-down"[tiab] OR "cooling"[tiab] OR "cold water immersion"[tiab] OR "cryotherapy"[tiab] OR "massage"[tiab] OR "foam roll*"[tiab] OR "compression garment*"[tiab] OR "active recovery"[tiab] OR "stretching"[tiab] OR "hydrotherapy"[tiab])<br>AND<br>("muscle damage"[tiab] OR "delayed onset muscle soreness"[tiab] OR DOMS[tiab] OR "creatine kinase"[tiab] OR performance[tiab] OR power[tiab] OR strength[tiab] OR jump[tiab] OR sprint[tiab] OR fatigue[tiab])<br>AND<br>(randomized controlled trial[pt] OR randomized[tiab] OR placebo[tiab])<br>NOT<br>("review"[pt] OR "meta-analysis"[pt] OR "animal"[tiab] OR "rat"[tiab] OR "mice"[tiab])**	**562**
**Web of Science**	**Web of Science Core Collection**	**January 24, 2026**	**TS=(("soccer" OR "football" OR "basketball" OR "volleyball" OR "rugby" OR "handball" OR "hockey" OR "lacrosse" OR "team sport*" OR "futsal") AND ("recovery" OR "cooling" OR "cold water immersion" OR "cryotherapy" OR "massage" OR "foam roll*" OR "compression garment*" OR "active recovery" OR**	**644**

Database	Platform	Search date	Full search strategy	Records retrieved
			"stretching") AND ("muscle damage" OR "DOMS" OR "creatine kinase" OR "performance" OR "fatigue") AND ("randomized" OR "trial" OR "RCT"))<br>NOT TI=("review" OR "meta-analysis" OR "animal" OR "rat" OR "mouse")	
SPORTDiscus	SPORTDiscus via EBSCOhost	January 24, 2026	(TI("soccer" OR "football" OR "basketball" OR "volleyball" OR "rugby" OR "handball" OR "hockey" OR "lacrosse" OR "team sport*" OR "futsal") OR AB("soccer" OR "football" OR "basketball" OR "volleyball" OR "rugby" OR "handball" OR "hockey" OR "lacrosse" OR "team sport*" OR "futsal"))<br>AND<br>(TI("recovery" OR "cooling" OR "cold water immersion" OR "cryotherapy" OR "massage" OR "foam roll*" OR "compression garment*" OR "active recovery") OR AB("recovery" OR "cooling" OR "cold water immersion" OR "cryotherapy" OR "massage" OR "foam roll*" OR "compression garment*" OR "active recovery"))<br>AND<br>(TI(randomized OR trial OR RCT) OR AB(randomized OR trial OR RCT))<br>NOT<br>(TI(review OR animal) OR DE "REVIEWS")	416
Cochrane Library	Cochrane Library	January 24, 2026	("soccer" OR "football" OR "basketball" OR "volleyball" OR "rugby" OR "hockey" OR "team sport")<br>AND<br>("recovery" OR "cooling" OR "massage" OR "compression" OR "stretching")<br>AND<br>("muscle damage" OR "performance")	829
Scopus	Scopus	January 24, 2026	TITLE-ABS-KEY(("soccer" OR "football" OR "basketball" OR "volleyball" OR "rugby" OR "hockey" OR "team sport*") AND ("recovery" OR "cold water immersion" OR	635

Database	Platform	Search date	Full search strategy	Records retrieved
			**"massage" OR "compression garment*" OR "active recovery") AND ("muscle damage" OR "performance") AND (randomized OR trial)) AND NOT TITLE-ABS-KEY(review OR animal OR rat) AND (LIMIT-TO(DOCTYPE, "ar"))**	

Abbreviations. TI = title; AB = abstract; TS = topic; DE = descriptor term; TITLE-ABS-KEY = title, abstract, and keywords.

Note. This table reproduces the database-specific search strategies as documented in the review workflow record and is intended for reporting in the supplementary materials.

Supplementary Table S2. Full-text articles excluded after eligibility assessment.

Reasons for exclusion for full-text reports assessed for eligibility but not included in the review.

Study	Article title	Primary reason for exclusion
**Pinto 2016**	**Photobiomodulation therapy (PBMT) improves performance and accelerates recovery of high-level Rugby players in field test: A randomized, crossover, double-blind, placebo-controlled clinical study**	**Intervention not eligible**
**Ravier 2018**	**Benefits of Compression Garments Worn During Handball-Specific Circuit on Short-Term Fatigue in Professional Players**	**Intervention not eligible**
**Abt 2011**	**The effects of a constant sprint-to-rest ratio and recovery mode on repeated sprint performance**	**Study design not eligible**
**Pournot 2011**	**Short term effects of various water immersions on recovery from exhaustive intermittent exercise**	**Study design not eligible**
**Skein 2013**	**The Effect of Overnight Sleep Deprivation After Competitive Rugby League Matches on Postmatch Physiological and Perceptual Recovery**	**Study design not eligible**
**Ribeiro 2017**	**Effect of Whole-Body Cryotherapy on Markers of Muscle Damage and Inflammation in Professional Soccer Players**	**Study design not eligible**
**Abdulkader 2025**	**Impact of self-myofascial release before and during complex training on creatine kinase and DOMS in football players**	**Insufficient or unusable data**
**Shimi 2016**	**The effect of time of day and recovery type after a football game on muscle damage and performance in anaerobic tests on young soccer players**	**Insufficient or unusable data**
**Gustafsson 2025**	**Cold- and hot-water immersion are not more effective than placebo for the recovery of physical performance and training adaptations in national level soccer players**	**Insufficient or unusable data**
**Micheletti 2019**	**A New Mathematical Approach to Explore the Post-exercise Recovery Process and Its Applicability in a Cold Water Immersion Protocol**	**Insufficient or unusable data**
**Alexander 2022**	**Utilisation of performance markers to establish the effectiveness of cold-water immersion as a recovery modality in elite football**	**Insufficient or unusable data**
**Jill Alexander 2022**	**Effects of contemporary cryo-compression on post-training performance in elite academy footballers**	**Insufficient or unusable data**
**Rupp 2012**	**The effect of cold water immersion on 48-hour performance testing in collegiate soccer players**	**Insufficient or unusable data**

Study	Article title	Primary reason for exclusion
Kavuran 2024	Investigation of the Effect of Restricted Environmental Stimulation Technique (Floatation-REST) in Floatation Tank on Physical, Physiological, and Psychological Recovery Parameters of Football Players	Insufficient or unusable data
Pavin 2018	Can compression stockings reduce the degree of soccer match-induced fatigue in females?	Insufficient or unusable data
Juliff 2014	Influence of contrast shower and water immersion on recovery in elite netballers	Insufficient or unusable data
Krueger 2019	The physiological effects of daily cold-water immersion on 5-day tournament performance in international standard youth field-hockey players	Insufficient or unusable data
Skein 2025	Cold-water recovery between bouts of simulated rugby sevens matches in the heat	Insufficient or unusable data
Hamlin 2012	Effect of compression garments on short-term recovery of repeated sprint and 3-km running performance in rugby union players	Insufficient or unusable data
Bouzid 2018	Faster physical performance recovery with cold water immersion is not related to lower muscle damage level in professional soccer players	Insufficient or unusable data
Sajadian 2017	Comparison of effects of active recovery and deep water running on soccer players' indices of muscular damage	Insufficient or unusable data
King 2009	The effects of recovery interventions on consecutive days of intermittent sprint exercise	Insufficient or unusable data
Pointon 2012	Cold water immersion recovery following intermittent-sprint exercise in the heat	Insufficient or unusable data
Babak 2021	Experience of cold-water immersion on recovery efficiency after soccer match	Insufficient or unusable data
Bouchiba 2022	Cold Water Immersion Improves the Recovery of Both Central and Peripheral Fatigue Following Simulated Soccer Match-Play	Insufficient or unusable data
Nasser 2023	Cold water immersion after a soccer match: Does the placebo effect occur?	Insufficient or unusable data
Pernigoni 2025	Investigating Recovery Strategies to Optimize Performance and Well-being in Basketball	Insufficient or unusable data
Pointon 2012	Cold Water Immersion Recovery after Simulated Collision Sport Exercise	Insufficient or unusable

Study	Article title	Primary reason for exclusion
		data
Atkins 2020	Lower-body compression garments worn following exercise improves perceived recovery but not subsequent performance in basketball athletes	Insufficient or unusable data
Russell 2016	The effects of a single whole body cryotherapy exposure on physiological, performance and perceptual responses of professional academy soccer players following repeated sprint exercise	Insufficient or unusable data
von Stengel 2018	Effect of deep oscillation as a recovery method after fatiguing soccer training: A randomized cross-over study	Insufficient or unusable data
Esteves 2025	Effects of Pneumatic Compression and Manual Massage on Recovery and Performance in Elite Brazilian Under-20 Soccer Players: A Crossover Trial	Insufficient or unusable data
Clifford 2017	Cryotherapy Re-Invented: Application of Phase Change Material for Recovery in Elite Soccer	Insufficient or unusable data
Taisuke 2009	A COMPARISON OF POST-MATCH RECOVERY STRATEGIES IN YOUTH SOCCER PLAYERS	Outcome not eligible
Kositsky 2020	The Effects of Cold Water Immersion on the Recovery of Drop Jump Performance and Mechanics: A Pilot Study in Under-20 Soccer Players	Outcome not eligible
Alexandre 2021	Effect of Transcranial Direct Current Stimulation on Professional Female Soccer Players' Recovery Following Official Matches	Outcome not eligible
Aloulou 2020	Effect of an Innovative Mattress and Cryotherapy on Sleep after an Elite Rugby Match	Outcome not eligible
Avina 2016	Post-exercise cold water immersion improves intermittent high-intensity exercise performance in normothermia	Outcome not eligible
Barry 2023	No effect of repeated post-resistance exercise cold or hot water immersion on in-season body composition and performance responses in academy rugby players: a randomised controlled cross-over design	Outcome not eligible
Beaven 2013	Electrostimulation's Enhancement of Recovery During a Rugby Preseason	Outcome not eligible
Brown 2022	Custom-Fitted Compression Garments Enhance Recovery From Muscle Damage in Rugby Players	Outcome not eligible
Buoite Stella 2024	The Acute Effects of Cold Water Immersion and Percussive Massage Therapy on Neuromuscular Properties and Muscle Soreness after Exercise in Young Male Soccer Players	Outcome not eligible

Study	Article title	Primary reason for exclusion
**Brownstein 2019**	**The Effect of Phase Change Material on Recovery of Neuromuscular Function Following Competitive Soccer Match-Play**	**Outcome not eligible**
**Chaiyakul 2021**	**Effects of Delayed Cold Water Immersion after High-Intensity Intermittent Exercise on Subsequent Exercise Performance in Basketball Players**	**Outcome not eligible**
**Chaiyakul 2025**	**Effects of combined cold-water immersion and Swedish massage on muscle strength, fatigue, blood lactate levels, and exercise performance in male team sport athletes**	**Outcome not eligible**
**Chow 2018**	**Differential effects of post-exercise ice water immersion and room temperature water immersion on muscular performance, vertical jump, and agility in amateur rugby players: A randomized controlled trial**	**Outcome not eligible**
**Getto 2013**	**Comparison of Active Recovery in Water and Cold-Water Immersion After Exhaustive Exercise**	**Outcome not eligible**
**Marqués-Jiménez 2018**	**Influence of different types of compression garments on exercise-induced muscle damage markers after a soccer match**	**Outcome not eligible**
**Duffield 2008**	**The Effects of Compression Garments on Intermittent Exercise Performance and Recovery on Consecutive Days**	**Outcome not eligible**
**Leal Junior 2009**	**Effect of 830 nm low-level laser therapy applied before high-intensity exercises on skeletal muscle recovery in athletes**	**Outcome not eligible**
**Ahokas 2023**	**A post-exercise infrared sauna session improves recovery of neuromuscular performance and muscle soreness after resistance exercise training**	**Outcome not eligible**
**Rey 2012**	**The effect of recovery strategies on contractile properties using tensiomyography and perceived muscle soreness in professional soccer players**	**Outcome not eligible**
**Rey 2017**	**The Effects of Foam Rolling as a Recovery Tool in Professional Soccer Players**	**Outcome not eligible**
**Vahdat 2014**	**Effects of Different Recovery Methods on Muscle Damage and Inflammation after Exhaustive Exercise**	**Outcome not eligible**
**Pesenti 2020**	**The effect of cold water immersion on pain, muscle recruitment and postural control in athletes**	**Outcome not eligible**
**Fenemor 2022**	**Small Performance Effects of a Practical Mixed-Methods Cooling Strategy in Elite Team Sport Athletes**	**Outcome not eligible**
**Crowther 2017**	**Effects of various recovery strategies on repeated bouts of simulated intermittent activity**	**Outcome not eligible**

Study	Article title	Primary reason for exclusion
**Tavares 2018**	**The Effects of Chronic Cold Water Immersion in Elite Rugby Players**	**Outcome not eligible**
**Bieuzen 2012**	**Recovery After High-Intensity Intermittent Exercise in Elite Soccer Players Using VEINOPLUS Sport Technology for Blood-Flow Stimulation**	**Outcome not eligible**
**Garcia 2016**	**Cold Water Immersion is Acutely Detrimental but Increases Performance Post-12h in Rugby Players**	**Outcome not eligible**
**García-Concepción 2015**	**Efficacy of different recovery strategies in elite football players**	**Outcome not eligible**
**Chow 2017**	**Effects of postexercise ice-water and room-temperature water immersion on the sensory organization of balance control and lower limb proprioception in amateur rugby players**	**Outcome not eligible**
**Gill 2006**	**Effectiveness of post-match recovery strategies in rugby players**	**Outcome not eligible**

Abbreviations. PBMT = photobiomodulation therapy.

Supplementary Table S3. League table for CK in the acute recovery phase (0-24 h).

Note. Lower triangle: results from network meta-analysis (column vs row). Upper triangle: results from direct comparisons (row vs column).

	Active recovery	Cold water	CON	LEDT	Placebo LEDT	Stretching	TWI
**Active recovery**	—	**0.04 [-2.09, 2.18]**	.	.	.	.	.
**Cold water**	**0.04 [-2.09, 2.18]**	—	**-1.12 [-2.23, 0.00]**	**-1.09 [-3.42, 1.25]**	**-0.24 [-2.55, 2.07]**	.	.
**CON**	**-1.07 [-3.48, 1.34]**	**-1.12 [-2.23, 0.00]**	—	.	.	.	.
**LEDT**	**-1.04 [-4.20, 2.12]**	**-1.09 [-3.42, 1.25]**	**0.03 [-2.56, 2.62]**	—	**0.84 [-1.48, 3.17]**	.	.
**Placebo LEDT**	**-0.20 [-3.34, 2.95]**	**-0.24 [-2.55, 2.07]**	**0.87 [-1.69, 3.43]**	**0.84 [-1.48, 3.17]**	—	.	.
**Stretching**	**0.04 [-2.09, 2.18]**	**-0.00 [-2.13, 2.13]**	**1.12 [-1.29, 3.52]**	**1.09 [-2.08, 4.25]**	**0.24 [-2.90, 3.39]**	—	.
**TWI**	**0.04 [-3.02, 3.11]**	**0.00 [-2.19, 2.19]**	**1.12 [-1.34, 3.57]**	**1.09 [-2.12, 4.29]**	**0.24 [-2.94, 3.43]**	**0.00 [-3.06, 3.06]**	—

Abbreviations. CON = control; LEDT = light-emitting diode therapy; TWI = thermoneutral water immersion. Negative values favor the column-defining intervention for CK reduction.

Supplementary Table S4. League table for CK in the delayed recovery phase (48-72 h).

Note. Lower triangle: results from network meta-analysis (column vs row). Upper triangle: results from direct comparisons (row vs column).

	**Active recovery**	**Cold water**	**CON**	**Stretching**	**TWI**
**Active recovery**	**—**	**0.36 [-1.64, 2.37]**	.	**-1.03 [-3.04, 0.99]**	.
**Cold water**	**0.36 [-1.64, 2.37]**	**—**	**-0.87 [-2.37, 0.63]**	**-1.39 [-3.41, 0.63]**	**-1.10 [-3.20, 0.99]**
**CON**	**-0.51 [-3.01, 1.99]**	**-0.87 [-2.37, 0.63]**	**—**	.	.
**Stretching**	**-1.03 [-3.04, 0.99]**	**-1.39 [-3.41, 0.63]**	**-0.52 [-3.04, 2.00]**	**—**	.
**TWI**	**-0.74 [-3.64, 2.16]**	**-1.10 [-3.20, 0.99]**	**-0.23 [-2.81, 2.34]**	**0.29 [-2.62, 3.20]**	**—**

Abbreviations. CON = control; TWI = thermoneutral water immersion. Negative values favor the column-defining intervention for CK reduction.

Supplementary Table S5. League table for CMJ in the acute recovery phase (0-24 h).

Note. Lower triangle: results from network meta-analysis (column vs row). Upper triangle: results from direct comparisons (row vs column).

	Active recovery	Cold water	CON	Dry exercise	Electrostimulation	Foam rolling	Massage	Multimodal recovery	Stretching	TWI	Water exercise
**Active recovery**	—	0.15 [-0.72, 1.02]	0.94 [0.05, 1.84]	.	.	.	.	.	0.07 [-0.80, 0.94]	.	.
**Cold water**	0.56 [-0.09, 1.20]	—	-0.10 [-0.46, 0.25]	.	.	.	0.25 [-0.53, 1.03]	.	-0.09 [-0.95, 0.78]	0.86 [-0.18, 1.90]	.
**CON**	0.52 [-0.13, 1.17]	-0.04 [-0.38, 0.31]	—	-0.21 [-1.21, 0.80]	0.09 [-0.91, 1.10]	.	-0.30 [-1.08, 0.48]	0.01 [-1.00, 1.01]	.	.	-0.02 [-1.03[illegible] 0.98]
**Dry exercise**	0.32 [-0.88, 1.51]	-0.24 [-1.30, 0.82]	-0.21 [-1.21, 0.80]	—	0.30 [-0.71, 1.31]	.	.	.	.	.	0.18 [-0.82[illegible] 1.19]
**[illegible]lectrostimulation**	0.61 [-0.58, 1.81]	0.06 [-1.01, 1.12]	0.09 [-0.91, 1.10]	0.30 [-0.71, 1.31]	—	.	.	.	.	.	-0.11 [-1.1[illegible] 0.89]
**Foam rolling**	0.09 [-1.21, 1.39]	-0.47 [-1.76, 0.83]	-0.43 [-1.75, 0.89]	-0.22 [-1.88, 1.44]	-0.52 [-2.18, 1.14]	—	.	.	0.18 [-0.83, 1.19]	.	.
**Massage**	0.51 [-0.40, 1.43]	-0.04 [-0.74, 0.65]	-0.01 [-0.70, 0.69]	0.20 [-1.02, 1.42]	-0.10 [-1.32, 1.12]	0.42 [-1.04, 1.88]	—	.	.	.	.
**Multimodal recovery**	0.53 [-0.67, 1.73]	-0.03 [-1.09, 1.04]	0.01 [-1.00, 1.01]	0.21 [-1.21, 1.64]	-0.08 [-1.50, 1.34]	0.44 [-1.22, 2.10]	0.02 [-1.21, 1.24]	—	.	.	.
**Stretching**	0.27 [-0.55, 1.09]	-0.29 [-1.11, 0.53]	-0.25 [-1.11, 0.60]	-0.05 [-1.37, 1.27]	-0.34 [-1.66, 0.98]	0.18 [-0.83, 1.19]	-0.24 [-1.30, 0.82]	-0.26 [-1.58, 1.06]	—	.	.
**TWI**	1.41 [0.19, 2.64]	0.86 [-0.18, 1.90]	0.89 [-0.20, 1.99]	1.10 [-0.39, 2.59]	0.80 [-0.69, 2.29]	1.32 [-0.34, 2.99]	0.90 [-0.35, 2.15]	0.88 [-0.60, 2.37]	1.14 [-0.18, 2.47]	—	.
**Water exercise**	0.50 [-0.70, 1.69]	-0.06 [-1.12, 1.00]	-0.02 [-1.03, 0.98]	0.18 [-0.82, 1.19]	-0.11 [-1.12, 0.89]	0.41 [-1.25, 2.07]	-0.02 [-1.24, 1.21]	-0.03 [-1.45, 1.39]	0.23 [-1.09, 1.55]	-0.92 [-2.40, 0.57]	—

Abbreviations. CON = control; TWI = thermoneutral water immersion. Positive values favor the column-defining intervention for CMJ improvement.

Supplementary Table S6. League table for CMJ in the delayed recovery phase (48-72 h).

Intervention	Active recovery	Cold water	CON	Foam rolling	Multimodal recovery	Stretching	TWI
**Active recovery**	**—**	**0.00 [-0.72, 0.72]**	**.**	**.**	**.**	**0.18 [-0.54, 0.89]**	**.**
**Cold water**	**-0.00 [-0.72, 0.72]**	**—**	**0.46 [0.01, 0.91]**	**.**	**.**	**0.18 [-0.54, 0.89]**	**1.00 [0.07, 1.93]**
**CON**	**0.46 [-0.38, 1.30]**	**0.46 [0.01, 0.91]**	**—**	**.**	**-0.13 [-1.01, 0.75]**	**.**	**.**
**Foam rolling**	**0.25 [-0.88, 1.38]**	**0.25 [-0.88, 1.38]**	**-0.21 [-1.42, 1.01]**	**—**	**.**	**-0.08 [-0.95, 0.80]**	**.**
**Multimodal recovery**	**0.33 [-0.89, 1.55]**	**0.33 [-0.66, 1.31]**	**-0.13 [-1.01, 0.75]**	**0.08 [-1.43, 1.58]**	**—**	**.**	**.**
**Stretching**	**0.18 [-0.54, 0.89]**	**0.18 [-0.54, 0.89]**	**-0.28 [-1.13, 0.56]**	**-0.08 [-0.95, 0.80]**	**-0.15 [-1.37, 1.07]**	**—**	**.**
**TWI**	**1.00 [-0.17, 2.17]**	**1.00 [0.07, 1.93]**	**0.54 [-0.49, 1.57]**	**0.75 [-0.72, 2.21]**	**0.67 [-0.68, 2.03]**	**0.82 [-0.35, 2.00]**	**—**

Note. Lower triangle: results from network meta-analysis (column vs row). Upper triangle: results from direct comparisons (row vs column).

Supplementary Table S7. League table for DOMS in the acute recovery phase (0-24 h).

Intervention	Active recovery	Cold water	CON	Foam rolling	Massage	Stretching	TWI	WBV + stretching
**Active recovery**	**—**	**0.28 [-2.59, 3.15]**	**.**	**.**	**.**	**0.00 [-2.87, 2.87]**	**.**	**.**
**Cold water**	**0.28 [-2.59, 3.15]**	**—**	**-1.36 [-2.37, -0.36]**	**.**	**-0.59 [-2.68, 1.49]**	**-0.28 [-3.15, 2.59]**	**-0.45 [-3.37, 2.47]**	**.**
**CON**	**-1.08 [-4.12, 1.96]**	**-1.36 [-2.36, -0.36]**	**—**	**.**	**4.72 [2.43, 7.01]**	**.**	**.**	**.**
**Foam rolling**	**0.22 [-3.88, 4.31]**	**-0.06 [-4.16, 4.03]**	**1.30 [-2.92, 5.51]**	**—**	**.**	**-0.22 [-3.13, 2.70]**	**.**	**.**
**Massage**	**1.34 [-2.10, 4.78]**	**1.06 [-0.83, 2.95]**	**2.42 [0.49, 4.36]**	**1.13 [-3.38, 5.63]**	**—**	**.**	**.**	**.**
**Stretching**	**0.00 [-2.87, 2.87]**	**-0.28 [-3.15, 2.59]**	**1.08 [-1.96, 4.12]**	**-0.22 [-3.13, 2.70]**	**-1.34 [-4.78, 2.10]**	**—**	**.**	**-0.24 [-3.14, 2.67]**
**TWI**	**-0.17 [-4.27, 3.92]**	**-0.45 [-3.37, 2.47]**	**0.91 [-2.18, 4.00]**	**-0.39 [-5.42, 4.64]**	**-1.51 [-4.99, 1.96]**	**-0.17 [-4.27, 3.92]**	**—**	**.**
**WBV + stretching**	**-0.24 [-4.32, 3.85]**	**-0.52 [-4.60, 3.57]**	**0.84 [-3.36, 5.05]**	**-0.46 [-4.57, 3.66]**	**-1.58 [-6.08, 2.92]**	**-0.24 [-3.14, 2.67]**	**-0.07 [-5.09, 4.95]**	**—**

Note. Lower triangle: results from network meta-analysis (column vs row). Upper triangle: results from direct comparisons (row vs column).

Supplementary Table S8. League table for DOMS in the delayed recovery phase (48-72 h).

Intervention	Active recovery	Cold water	CON	Foam rolling	Stretching	TWI	WBV + stretching
**Active recovery**	**—**	**0.59 [-1.61, 2.80]**	**.**	**.**	**-2.08 [-4.32, 0.16]**	**.**	**.**
**Cold water**	**0.59 [-1.61, 2.80]**	**—**	**-1.59 [-2.54, -0.63]**	**.**	**-2.67 [-4.94, -0.41]**	**-1.71 [-4.03, 0.61]**	**.**
**CON**	**-0.99 [-3.40, 1.41]**	**-1.59 [-2.54, -0.63]**	**—**	**.**	**.**	**.**	**.**
**Foam rolling**	**-1.40 [-4.59, 1.79]**	**-2.00 [-5.20, 1.21]**	**-0.41 [-3.75, 2.94]**	**—**	**-0.68 [-2.94, 1.59]**	**.**	**.**
**Stretching**	**-2.08 [-4.32, 0.16]**	**-2.67 [-4.94, -0.41]**	**-1.09 [-3.55, 1.38]**	**-0.68 [-2.94, 1.59]**	**—**	**.**	**0.77 [-1.48, 3.02]**
**TWI**	**-1.12 [-4.32, 2.08]**	**-1.71 [-4.03, 0.61]**	**-0.12 [-2.63, 2.38]**	**0.28 [-3.67, 4.24]**	**0.96 [-2.28, 4.20]**	**—**	**.**
**WBV + stretching**	**-1.31 [-4.49, 1.87]**	**-1.90 [-5.10, 1.29]**	**-0.32 [-3.65, 3.02]**	**0.09 [-3.10, 3.29]**	**0.77 [-1.48, 3.02]**	**-0.19 [-4.14, 3.76]**	**—**

Note. Lower triangle: results from network meta-analysis (column vs row). Upper triangle: results from direct comparisons (row vs column).

Supplementary Table S9. The PRISMA 2020 reporting checklist

The PRISMA 2020 reporting checklist

For checking that systematic review articles can be understood and used by everyone

	**Item Description**	**Location (or reason for not reporting)**
**Title and Abstract**		
1. Title	Identify the report as a systematic review.	Title page/title section.
2. Abstract	Include all items from the *PRISMA 2020 for Abstracts* checklist.	Structured Abstract: Background, Objective, Methods, Results, and Conclusion.
**Introduction**		
3. Rationale	Describe the rationale for the review in the context of existing knowledge.	Introduction, paragraphs 1-3.
4. Objectives	Provide an explicit statement of the objective(s) or question(s) the review addresses.	Introduction, final paragraph.
**Methods**		
5. Eligibility criteria	Specify the inclusion and exclusion criteria for the review and how studies were grouped for the syntheses.	Methods - Search strategy and eligibility criteria; Eligibility criteria.
6. Information sources	Specify all databases, registers, websites, organisations, reference lists and other sources searched or consulted to identify studies. Specify the date when each source was last searched or consulted.	Methods - Information sources and search strategy; Supplementary Table S1.
7. Search	Present the full search strategies for all databases, registers and websites, including any	Supplementary Table S1.

	filters and limits used.	
8. Selection Process	Specify the methods used to decide whether a study met the inclusion criteria of the review, including how many reviewers screened each record and each report retrieved, whether they worked independently, and, if applicable, details of automation tools used in the process.	Methods - Study selection.
9. Data collection process	Specify the methods used to collect data from reports, including how many reviewers collected data from each report, whether they worked independently, any processes for obtaining or confirming data from study investigators, and if applicable, details of automation tools used in the process.	Methods - Data extraction.
**10. Data Items**		
10a. Outcomes	List and define all outcomes for which data were sought. Specify whether all results that were compatible with each outcome domain in each study were sought (e.g. for all measures, time points, analyses), and if not, the methods used to decide which results to collect.	Abstract - Methods; Methods - Data extraction; Methods - Time-window aggregation and subnetwork management; Methods - Statistical analysis.
10b. Other Variables	List and define all other variables for which data were sought (e.g. participant and intervention characteristics, funding sources). Describe any assumptions made about any missing or unclear information.	Methods - Data extraction.
11. Risk of bias in individual studies	Specify the methods used to assess risk of bias in the included studies, including details of the tool(s) used, how many reviewers assessed each study and whether they worked independently, and if applicable, details of automation tools used in the process.	Methods - Risk of bias assessment.
12. Effect measures	Specify for each outcome the effect measure(s) (e.g. risk ratio, mean difference) used in the synthesis or presentation of results.	Methods - Statistical analysis.

**13. Synthesis Methods**		
13a. Deciding which studies were eligible for each synthesis	Describe the processes used to decide which studies were eligible for each synthesis (such as tabulating the study intervention characteristics and comparing against the planned groups for each synthesis described in item 5.	Methods - Time-window aggregation and subnetwork management.
13b. Data preparation methods	Describe any methods required to prepare the data for presentation or synthesis, such as handling of missing summary statistics, or data conversions.	Methods - Data extraction; Methods - Time-window aggregation and subnetwork management.
13c. Methods for tabulating or displaying results	Describe any methods used to tabulate or visually display results of individual studies and syntheses.	Methods - Statistical analysis; Results - Table 1, Table 2, Figure 2, Figures 3-5, and Supplementary Figures S1-S4.
13d. Synthesis methods	Describe any methods used to synthesize results and provide a rationale for the choice(s). If meta-analysis was performed, describe the model(s), method(s) to identify the presence and extent of statistical heterogeneity, and software package(s) used.	Methods - Statistical analysis.
13e. Methods for exploring heterogeneity	Describe any methods used to explore possible causes of heterogeneity among study results (e.g. subgroup analysis, meta-regression).	Methods - Statistical analysis; Results - Heterogeneity and inconsistency; Supplementary Figures S1-S4.
13f. Sensitivity analyses	Describe any sensitivity analyses conducted to assess robustness of the synthesized results.	Not conducted; no formal sensitivity analyses were performed because several networks contained limited numbers of studies and sparse comparisons.
14. Reporting bias assessment	Describe any methods used to assess risk of bias due to missing results in a synthesis (arising from reporting biases).	Methods - Statistical analysis; Results - Heterogeneity and inconsistency;

		Supplementary Figure S1.
15. Certainty assessment	Describe any methods used to assess certainty (or confidence) in the body of evidence for an outcome.	Not assessed; no formal certainty/confidence framework (e.g., GRADE or CINeMA) was applied.
**Results**		
**16. Study Selection**		
16a. Results of the search and selection process	Describe the results of the search and selection process, from the number of records identified in the search to the number of studies included in the review, ideally using a flow diagram.	Results - Study selection and characteristics; Figure 1.
16b. Excluded studies	Cite studies that might appear to meet the inclusion criteria, but which were excluded, and explain why they were excluded.	Results - Study selection and characteristics; Supplementary Table S2.
17. Study characteristics	Cite each included study and present its characteristics.	Results - Study selection and characteristics; Table 1.
18. Risk of bias in studies	Present assessments of risk of bias for each included study.	Results - Study selection and characteristics; Figure 2.
19. Results of individual studies	For all outcomes, present, for each study: (a) summary statistics for each group (where appropriate) and (b) an effect estimate and its precision (e.g. confidence/credible interval), ideally using structured tables or plots.	Results - Acute recovery phase (0-24 h); Delayed recovery phase (48-72 h); Table 2; Figures 4-5; Supplementary Tables S3-S8.
**20. Results of Synthesis**		
20a. Summary of studies	For each synthesis, briefly summarise the characteristics and risk of bias among contributing studies.	Results - Network geometry; Acute recovery phase (0-24 h); Delayed recovery phase (48-72 h); Table 2; Figure 3.

20b. Statistical results	Present results of all statistical syntheses conducted. If meta-analysis was done, present for each the summary estimate and its precision (e.g. confidence/credible interval) and measures of statistical heterogeneity. If comparing groups, describe the direction of the effect.	Results - Acute recovery phase (0-24 h); Delayed recovery phase (48-72 h); Table 2; Figures 4-5.
20c. Heterogeneity	Present results of all investigations of possible causes of heterogeneity among study results.	Results - Heterogeneity and inconsistency; Supplementary Figures S1-S4.
20d. Sensitivity analyses	Present results of all sensitivity analyses conducted to assess the robustness of the synthesized results.	Not applicable; no formal sensitivity analyses were conducted.
21. Risk of reporting biases in syntheses	Present assessments of risk of bias due to missing results (arising from reporting biases) for each synthesis assessed.	Results - Heterogeneity and inconsistency; Supplementary Figure S1.
22. Certainty of evidence	Present assessments of certainty (or confidence) in the body of evidence for each outcome assessed.	Not assessed; no formal certainty/confidence assessment was performed for the body of evidence.
**Discussion**		
**23. Discussion**		
23a. General interpretation of the results	Provide a general interpretation of the results in the context of other evidence.	Discussion - Principal findings; Mechanistic interpretation; Delayed recovery and the role of CWI.
23b. Limitations of included evidence	Discuss any limitations of the evidence included in the review.	Discussion - Limitations.
23c. Limitations of the review processes	Discuss any limitations of the review processes used.	Discussion - Limitations (including restriction to connected main networks

		and related review-process constraints).
23d. Implications	Discuss implications of the results for practice, policy, and future research.	Discussion - Clinical implications; Conclusion.
**Other Information**		
**24. Registration and Protocol**		
24a. Registration	Provide registration information for the review, including register name and registration number, or state that the review was not registered.	Methods - Protocol and reporting.
24b. Protocol	Indicate where the review protocol can be accessed, or state that a protocol was not prepared.	Methods - Protocol and reporting; Data availability.
24c. Amendments	Describe and explain any amendments to information provided at registration or in the protocol.	No protocol amendments were reported after OSF archiving.
25. Support	Describe sources of financial or non-financial support for the review, and the role of the funders or sponsors in the review.	Funding.
26. Competing Interests	Declare any competing interests of review authors.	Declaration of competing interest.
27. Availability of data, code, and other materials	Report which of the following are publicly available and where they can be found: template data collection forms; data extracted from included studies; data used for all analyses; analytic code; any other materials used in the review.	Data availability; Methods - Protocol and reporting; Supplementary Table S1; Supplementary Table S2.
